# An outpatient clinic for integrative mental health: Patient and treatment characteristics and health outcomes based on patient reported routine outcome monitoring data

**DOI:** 10.1192/j.eurpsy.2021.1041

**Published:** 2021-08-13

**Authors:** R. Hoenders, A. Bartels, S. Castelein, E. Visser, S. Booij

**Affiliations:** 1 Center For Integrative Psychiatry, Lentis, Groningen, Netherlands; 2 Psychiatry, Rgoc, University of Groningen, University Medical Center Groningen, Groningen, Netherlands; 3 Clinical Psychology And Experimental Psychopathology, University of Groningen, Groningen, Netherlands; 4 Lentis Research, Lentis, Groningen, Netherlands; 5 Department Of Psychiatry, University of Groningen, University Medical Center Groningen, Groningen, Netherlands

**Keywords:** routine outcome monitoring, integrative psychiatry, fundamental prognostic research, patient reported outcomes

## Abstract

**Introduction:**

There is an increasing interest in integrative (mental) health care and a growth in centers offering such services, but a paucity of research on patient characteristics, diagnosis, treatments offered, the effects of those treatments and patient satisfaction.

**Objectives:**

To examine the course of mental health outcomes in the context of the nature and quality of care of outpatients at a center for integrative psychiatry in the Netherlands, as well as relevant sociodemographic, clinical, and treatment-related moderators of this course.

**Methods:**

Baseline patient demographics, clinical and treatment characteristics of 537 patients with a completed care episode between 2012 and 2019 were assessed. Satisfaction and mental health treatment outcomes were examined using routine outcome monitoring and analyzed with multilevel intention-to-treat models.

**Results:**

Two thirds of patients were woman (median age 41 years), predominantly with a primary diagnosis of mood or anxiety disorder. Mean number of treatment sessions was 49 (SD=94) and total clinical time was 54 hours (SD=109). Mean treatment duration was 460 days (SD=407). Ninety percent of the sample filled out one or more assessment(s). Of the individuals with a baseline assessment, 50% completed a follow-up. Significant improvements in symptomatology, social functioning, interpersonal functioning, wellbeing, resilience and quality of life were found. Clinical and scientific interpretation, moderator analyses and patient satisfaction will be presented at the conference.

**Conclusions:**

Although no definite conclusions can be drawn due to the naturalistic design and missing data, especially at follow-up, patients seem to improve on all measured domains, including psychopathology, functioning and wellbeing.

